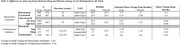# Comparing Apples to Oranges: A comparison on Efficacy data for monoclonal antibodies and cholinesterase inhibitors for treatment of Alzheimer’s Disease from FDA Medical and Statistical Reports

**DOI:** 10.1002/alz70859_104054

**Published:** 2025-12-26

**Authors:** Aishwarya Prasad, Anshu Arora, Arun Arora, Arif Khan

**Affiliations:** ^1^ Northwest Clinical Research Center, Bellevue, WA USA

## Abstract

**Background:**

Current Alzheimer’s literature suggests that monoclonal antibodies (mAbs) provide only modest benefit compared to cholinesterase inhibitors (AChEIs) and memantine. However, direct comparative data between these two drug classes remain limited. In this study, we seek to compare their efficacy based on the available evidence.

**Methods:**

We examined efficacy data from FDA archives (https://www.accessdata.fda.gov/scripts/cder/daf/index.cfm) through October 2024 to understand the risk‐benefit profile of mAbs and AChEIs. For efficacy analysis, we focused on five medications encompassing 11,122 subjects.

Our search targeted donanemab, lecanemab, aducanumab, donepezil, galantamine. We included all drugs that evaluated patients using the ADAS‐Cog scale as one of their efficacy outcome measures.

Memantine was excluded from analysis because it did not use ADAS‐Cog as a efficacy outcome measure.

**Results:**

Efficacy was reported in terms of difference of adjusted mean change in ADAS‐Cog from baseline to end of study. The difference between drug and placebo for AChEIs was ‐2.82 over an average duration of 25.5 weeks. For mAbs, the average difference was ‐1.08 over an average duration of 77.3 weeks.

**Conclusion:**

All drugs showed a statistically significant difference ADAS‐Cog scores relative to placebo. However, mAbs are tested and approved for Mild Cognitive Impairment (MCI) or mild dementia. On the other hand, AChEIs were tested and approved for moderate to severe AD.

Since these studies employed different versions of the ADAS‐Cog scale (e.g., ADAS‐Cog 11 vs. ADAS‐Cog 13), making direct comparisons of efficacy more difficult. Additional research is needed to fully assess the efficacy of these newer therapies compared to placebo.